# The Association between Delayed Gut Microbiota Maturity in Pre-Term Infants and the Feeding Intolerance—A Pilot Study

**DOI:** 10.3390/biomedicines12030539

**Published:** 2024-02-28

**Authors:** Ya-Chi Hsu, Ming-Chih Lin, Katharina Ardanareswari, Webiana Lowisia, Yi-Hsuan Lin, Yi-Jhen Chen, Cheng-Kuang Hsu, Yun-Chin Chung

**Affiliations:** 1 Division of Neonatology, Children’s Medical Center, Taichung Veterans General Hospital, Taichung 407219, Taiwan; syach721030@gmail.com (Y.-C.H.); mingclin@gmail.com (M.-C.L.); joyce750210@hotmail.com (Y.-H.L.); 2 Department of Food and Nutrition, Providence University, Taichung 43301, Taiwan; k.ardanareswari@gmail.com (K.A.); fwebiana@gmail.com (W.L.); 1060457@pu.edu.tw (Y.-J.C.); 3 Department of Food Technology, Faculty of Agricultural Technology, Soegijapranata Catholic University, Semarang 50234, Indonesia; 4 Department of Food Science, National Chiayi University, Chiayi 60004, Taiwan; ckhsu@mail.ncyu.edu.tw

**Keywords:** 16S rDNA sequencing, feeing intolerance, fecal microbiota, full-term infants, pre-term infants

## Abstract

This study compared gut (fecal) microbiota profiles between pre-term and full-term infants, assuming that pre-term infants without feeding intolerance would have gut microbiota similar to those of full-term infants. A total of 13 pre-term infants (gestational age < 37 weeks, birthweight ≤ 2500 g) and 10 full-term infants were included. The pre-term infants were assigned to the feeding tolerance (FT) group (n = 7) if their daily intake exceeded 100 mL/kg/day at two weeks after birth, or the feeding intolerance (FI) group (n = 6). Microbial DNA from weekly fecal samples was analyzed. The microbiota profiles of the pre-term infants and full-term infants were significantly different (*p* = 0.0001), as well as the FT and FI groups (*p* = 0.0009). The full-term group had more diversity, with higher concentrations of facultative anaerobes such as Bifidobacteriaceae and Lactobacteriaceae. The FT group’s gut microbiota matured over four weeks, with higher levels of digestion-related bacteria, while the FI group had more pathogens. In the FI group, a significant difference was observed between the first and second weeks, with no significant differences noted between the first week and the third or fourth weeks. The delay in the development of the pre-term infants’ gut microbiota may be associated with the FI.

## 1. Introduction

After birth, the colonization of the gut microbiota is influenced by organ maturity and external factors such as delivery method, antibiotic treatments, and choice of feeding method [1]. The time from birth to the establishment of a stable gut microbiome (at about three years of age) is an important period that affects the infant’s later health [2]. Compared to full-term infants, pre-term infants exhibit the relatively late establishment of the gut microbiota, as well as lower diversity [3]. The gastrointestinal tract of pre-term infants may contain low levels of anaerobes and be dominated by potentially pathogenic Enterobacteriaceae of the Proteobacteria phylum [4].

The digestive tract of a full-term infant is usually mature enough to digest and absorb breastmilk or formula, allowing for adequate nutrition. Conversely, pre-term infants have limited capacity to use food intake to support their growth due to their organ immaturity, gastrointestinal malabsorption, and poor digestive capacity. For example, although breast milk is the ideal nutrition for infants, the lactose hydrolysis enzyme system in the intestines of pre-term infants is not yet fully developed, meaning that pre-term infants may not be ready to digest proteins [5]. The protease activity itself does not differ between pre-term infants and full-term infants, but the pH of the gastrointestinal tract of pre-term infants is too high, affecting the activity of pepsin [6,7]. These digestive functions may not only relate to the maturation of the gastrointestinal tract but also to the gut microbiota [8].

The analysis of the gut microbiota of pre-term infants is influenced by many biological and non-biological factors. Delays in meconium passage or small amounts of stool from pre-term infants with very low birthweights often lead to the insufficient availability of samples for gut microbiota analysis. With the novel development of next-generation sequencing (NGS) techniques, such sampling and identification constraints can be overcome. Using this technique, it is possible to obtain the genetic information of environmental microbial communities quickly and accurately. We hypothesized that the limited digestive capacity of pre-term infants might be due to the delayed maturation of gut microbiota, which results in feeding intolerance (FI). FI is a well-known phenomenon in neonatal intensive care units (NICUs) and constitutes a benign condition related to the immaturity of gastrointestinal function. Some researchers define FI in premature infants using outcome measurements including the failure to reach full enteral feeding during a specific timeframe or the number of interruptions or delays in the process of reaching full enteral feeding [9]. Feeding problems are one of the main factors that lead to the prolonged hospitalization of premature infants in NICUs, which is related to morbidities in pre-term infants [10]. Therefore, we sought to determine the relationship between the gut microbiota composition and feeding conditions in infants.

We conducted a prospective cohort study from January to May in 2021, in which 13 pre-term infants and 10 full-term infants were enrolled. We divided the 13 pre-term infants into two groups: the feeding tolerance (FT) group and the feeding intolerance (FI) group. Stool samples were collected each week in the first month of life and the gut microbiota were analyzed using 16S rRNA (V3–V4 region) gene sequences. We compared weekly changes in the gut microbiota of pre-term infants and full-term infants, as well as differences in gut microbiota between the FT group and the FI group.

## 2. Materials and Methods

### 2.1. Ethical Approval

This study was approved by the Institutional Review Board of Taichung Veterans General Hospital (TVGH) No. CF18180A. The guardians were aware of the scope of this research and gave their consent.

### 2.2. Subjects

The subjects of this study were 13 pre-term infants and 10 full-term infants born in the Children’s Medical Center of the Taichung Veterans General Hospital (TVGH) during the period from January to May 2021. The pre-term infants included in this study were classified as low-birthweight infants, defined as those with a gestational age of less than 37 weeks and a birthweight of less than 2500 g. Ten full-term healthy infants were included as the control group. Infants with severe chromosomal abnormalities or congenital abnormalities were excluded. The demographic characteristics of the enrolled infants were gathered (Supplementary Materials Table S1), including the gestational age, birthweight, type of delivery, days using antibiotics or use of probiotics, prokinetic agent, an H2 blocker, and the diagnosis of necrotizing enterocolitis (NEC). All pre-term infants had non-invasive ventilator support during the first month of life. The full-term infants received routine care in the nursery and had their daily diets recorded. The flow chart of participant recruitment is included in Supplementary Materials Figure S1.

### 2.3. Fecal Collection

The infants’ feces (1 g) were collected in sterile stool collection tubes weekly during the first month of life. The tubes were immediately frozen in a −20 °C freezer. A total of 92 samples were gathered. All collected samples were then transferred for DNA extraction to the Department of Food and Nutrition, Providence University.

### 2.4. Feeding Tolerance Assessment

Parenteral nutrition was given to all pre-term participants on the first day of life (DOL), which was followed by trophic feeding as soon as possible. The feeding advancement was individualized according to the clinician’s discretion. The breast milk or formula intake was recorded daily. When the enteral feeding intake reached 100 mL/kg/day, the parenteral nutrition was discontinued, and the fortified feedings were commenced. According to the feeding amounts at 14 DOL, the pre-term infants were divided into 2 groups: the FT group who could receive more than 100 mL/kg/day, and the FI group who could not.

### 2.5. Gut Microbiota Analysis

Genomic DNA from feces was extracted according to the instructions of the QIAamp DNA Stool Mini Kit (QIAGEN, Germantown, MD, USA). The quality of the extracted DNA was checked for its concentration and purity using spectrophotometry and 1% agarose gels, respectively.

Sequencing primers with the barcodes 341F (CCTACGGGNGGCWGCAG) and 805R (GACTACHVGGGTATCTAATCC) were used to amplify the V3–V4 section of 16S ribosomal DNA. A polymerase chain reaction (PCR) was performed in a 25 µL mixture containing 0.5 µL KAPA High-Fidelity PCR Master Mix (KAPA Biosystems, Wilmington, MA, USA), 0.5 µM of each primer mentioned above, and 1 ng of template DNA. After 3 min of initial denaturation at 95 °C, 30 cycles of the PCR thermal cycle were set to denaturation at 95 °C for 30 s, annealing at 57 °C for 30 s, elongation at 72 °C for 30 s, and a 5 min extension at 72 °C. For quantification and qualification, the PCR products were mixed with SYB Green and electrophorized on 2% agarose gels. DNA bands 450–500 bp in size were selected and purified using the QIAquick Gel Extraction Kit (QIAGEN, Germantown, MD, USA). Sequencing libraries were created using the Truseq Nano DNA Library Prep Kit (Illumina, San Diego, CA, USA). After being checked with a Qubit 2.0 Fluorometer (Thermo Scientific, Waltham, MA, USA) and an Agilent Bioanalyzer 2100 system, the libraries were sequenced by an Illumina MiSeq platform of 300 bp paired end reads generated by Genomics, BioSci and Tech Co. (New Taipei City, Taiwan).

### 2.6. Data Analysis

Paired end reads (2 × 300 bp) were trimmed [11] and demultiplexed by an in-house script. Sequences from both ends of the 341F–805R primers were trimmed using Cutadapt with the following criteria: read length ≥150 bp and error rate 0.1 as a default [12]. DADA2 (v1.12) was subsequently used to perform the preprocessing, which included filtering out noisy sequences (denoising), merging paired end reads, and removing chimera to extract amplicon sequence variants (ASV) [13]. The identified ASV sequences were annotated with a QIIME2 naive Bayes classifier (v2019.10) using the SILVA 132 99% identity as a reference [14,15]. A Venn diagram (v1.6.17) was used to analyze both the common and unique information between the different samples. The results of the species annotation were interactively shown using KRONA (v2.7.1) [16]. The alpha diversity was analyzed by a species richness estimator (Chao1, observed species, Good’s coverage, and Fisher’s alpha) and a species evenness estimator (Shannon, Simpson, and ENSPIE). The non-metric multidimensional scaling (NMDS) ordination technique was employed in conjunction with correlation analysis and geographical information systems (GIS) to analyze huge datasets in order to identify spatial relationships [17]. Linear discriminant analysis effect size (LEfSe) analysis mines biomarkers between two or more groups with effect sizes [18]. The analysis of similarities (ANOSIM) was conducted using the Vegan: Community Ecology Package v2.5-7 and R Package v2.5.7n [19]. SPSS 26.0 software was used to perform one-way ANOVA when the data fulfilled the normal distribution and homogeneity assumptions to find the significant difference between groups (*p* < 0.05).

## 3. Results

### 3.1. Demographic Characteristics

The demographic characteristics of the 10 full-term infants and 13 pre-term infants included in the study are described below. No full-term infants were admitted to the hospital or given antibiotics during the study period. The full-term infants received routine nursery care and had their daily diets recorded. The gestational age of the full-term group was 38.09 ± 1.34 weeks, with a birthweight of 3133 ± 239.46 g. Based on their intake on the 14th DOL, 6 of the pre-term infants were included in the FI group and 7 of them in the FT group. All the full-term and pre-term infants were exclusively fed with breastmilk during the first two weeks. Additionally, five full-term infants, and four pre-term infants (three in the FT group) received mixed feeding with additional formula afterwards. Comparing the two groups of pre-term infants, the mean gestational ages were 29.33 ± 1.95 and 32.67 ± 1.74 weeks; the mean birthweights were 1284.17 ± 270.56 and 1681.43 ± 364.94 g, respectively. Additionally, the number of antibiotic days in the first two weeks of life was 8.83 ± 4.54 and 3.57 ± 1.51 days, respectively. There were significant differences in the gestational age and days of antibiotic use between the infants, and the *p*-values were 0.008 and 0.035, respectively. None of the pre-term infants had culture-proved sepsis, NEC, probiotic supplements, prokinetic agents, or H2-blocker medication during the study period. All the detailed characteristics of the full-term and pre-term infants are listed in the Supplementary Materials Table S1.

### 3.2. Gut Microbiome Profiles of the Full-Term and Pre-term Groups

**Figure 1:**
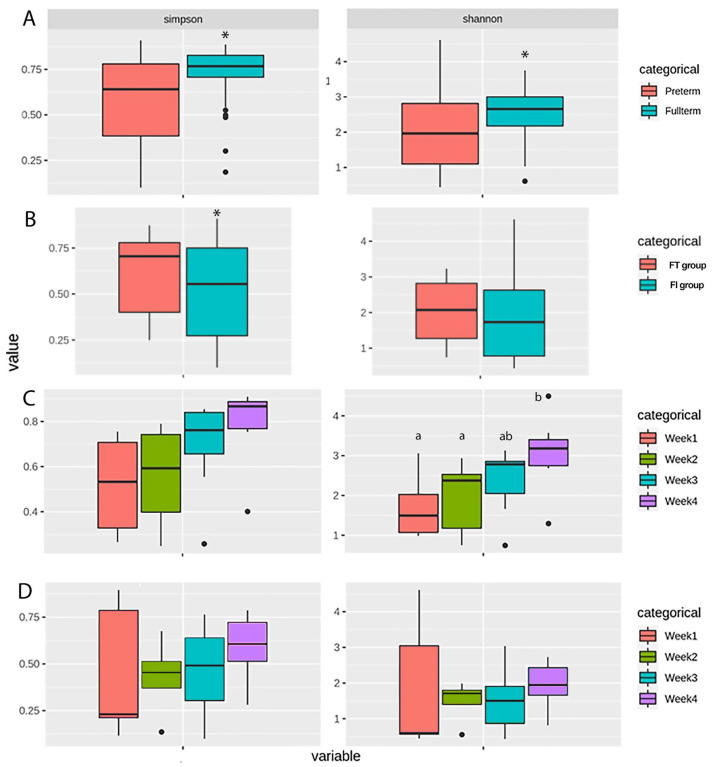
Alpha diversity measured by the Shannon and Simpson indices of (**A**) pre-term and full-term groups, (**B**) FT and FI groups, (**C**) FT group over four weeks, and (**D**) FI group over four weeks. * Significant difference between groups (*p* < 0.05); ^ab^ different letters indicate a significant difference among weeks (*p* < 0.05).

**Figure 2:**
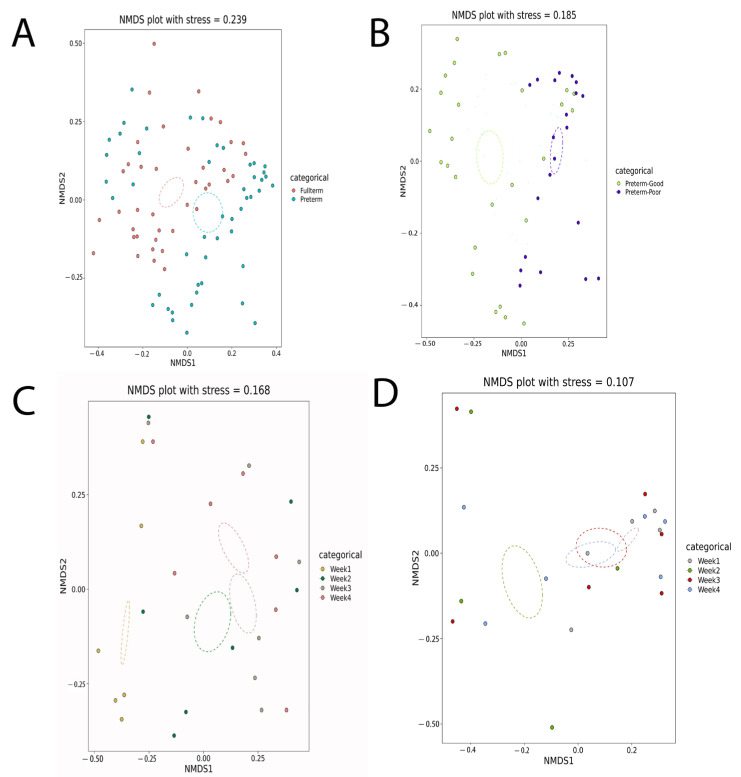
NMDS graph of (**A**) pre-term and full-term infants, (**B**) FT and FI pre-term infants, (**C**) FT pre-term infants over four weeks, and (**D**) FI pre-term infants over four weeks.

Based on the ANOSIM analysis, the overall fecal microbiomes of the full-term and pre-term groups were statistically different (*p* = 0.0001, Table 1). The alpha diversity, measured by the Shannon and Simpson indices, was significantly higher for full-term infants than for pre-term infants (Figure 1A). The NMDS analysis for beta diversity also showed a clear cluster separation between full-term and pre-term infants (Figure 2A).

**Figure 3:**
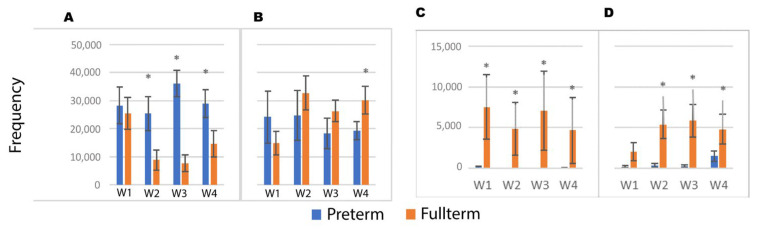
Absolute abundance of major phyla in full-term and pre-term groups. (**A**) Proteobacteria, (**B**) Firmicutes, (**C**) Bacteroidetes, (**D**) Actinobacteria. Wn represents the nth week. * Significant difference among groups (*p* < 0.05).

The phyla Proteobacteria (Figure 3) was decreased in full-term infants starting from week two but remained stable in pre-term infants. The abundance of Proteobacteria was significantly higher in pre-term infants throughout almost the entire study period, while levels of Actinobacteria and Bacteroidetes were higher in full-term infants (Figure 3). Firmicutes was stable in full-term infants from week two when it was consistently higher than in the pre-term infants. In addition, Bacteroidetes was consistently higher in full-term infants than in pre-term infants (Figure 3). Aerobes Moraxellaceae were some of the major families in the pre-term infants, but they were found in quantities of less than 1% in the full-term infants (Supplementary Materials Table S2).

### 3.3. Overall Microbiome Profiles of the FT and FI Groups and the Changes over Four Weeks

The overall fecal microbiomes of the FT and FI groups were significantly different (*p* = 0.0009, Table 1). Over four weeks, the pre-term infants with FT exhibited significant differences between week one and the rest of the study period. However, in the FI group, a significant difference was only detected between the first and second weeks (Table 1).

A significantly higher alpha diversity was also exhibited in the FT group compared to the FI group (Figure 1B) according to the Simpson index. When the alpha diversity of each week was compared, it was found that during the first four weeks of life, the gut microbiota of the FT group showed significant differences between the first week and the rest of the study period. However, in the FI group, significant differences were only detected between the first and second week (Figure 1C,D).

Furthermore, the beta diversity (NMDS) analysis showed a significant difference between the FT and FI groups (Figure 2B). In line with the ANOSIM results, throughout the four-week period, the gut microbiome for each week in the FT group formed separate clusters (Figure 2C). However, in the FI group, although the gut microbiome in week one was separated from the rest of the weeks, weeks two, three, and four were clustered together (Figure 2D).

**Figure 4:**
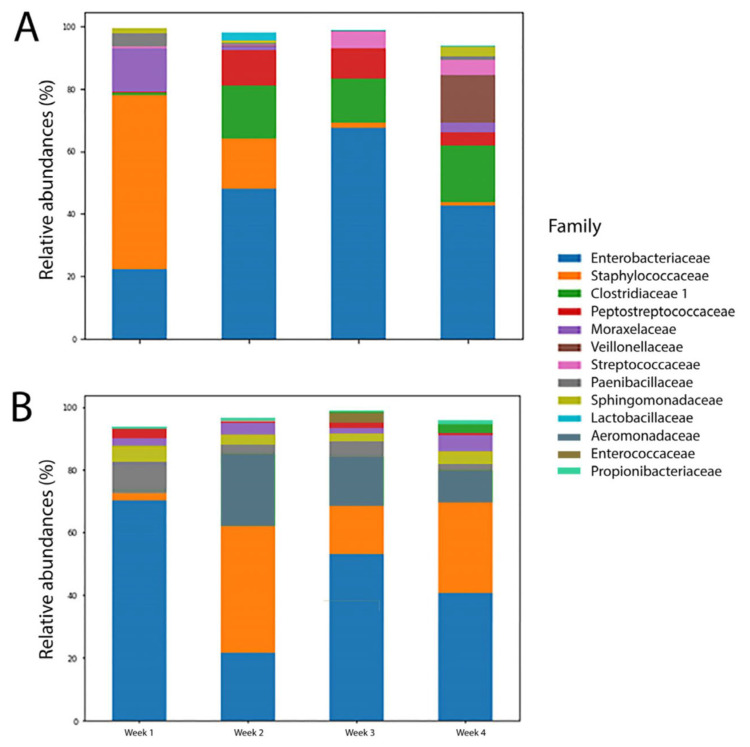
Top 10 gut microbiota families of (**A**) the FT group and (**B**) the FI group over four weeks.

As shown in Figure 4, the aerobe Moraxellaceae was detected in the first week but practically disappeared thereafter. Staphylococcaceae also decreased in the FT group. Additionally, the levels of obligate anaerobes such as Peptostreptococcaceae and Clostridiaceae started to rise from week two. Such a development was not observed in the FI group, where the aerobe Moraxellaceae was consistently placed in the top 10 list throughout the four-week period.

**Figure 5:**
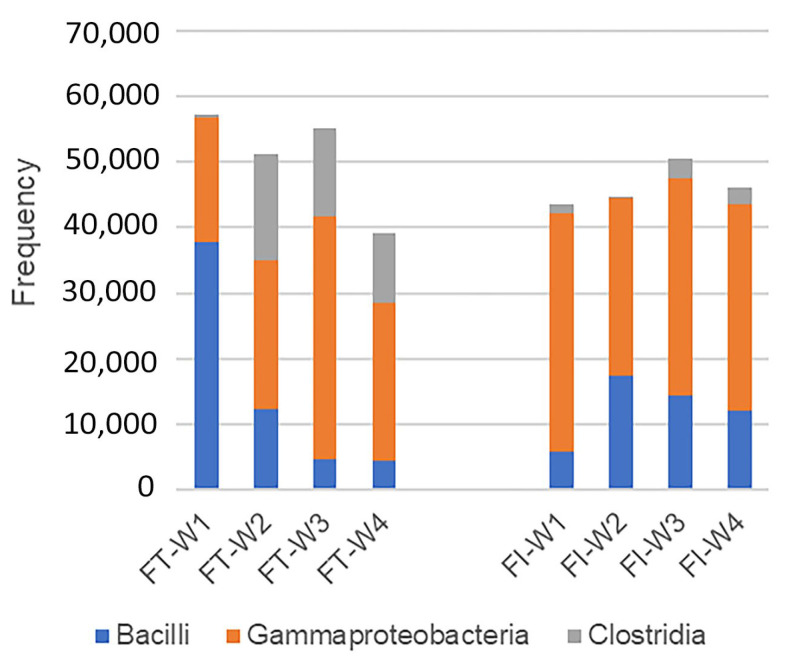
Dynamics of bacilli, Gammaproteobacteria, and clostridia in the FT and FI groups.

In addition, bacilli were phased out and replaced by increasing amounts of clostridia starting from week two in the FT group (Figure 5). In the FI group, however, there was minimal change in total bacilli, Gammaproteobacteria, and clostridia.

### 3.4. Biomarkers in Full-Term and Pre-term Infants: FT and FI Groups

**Figure 6:**
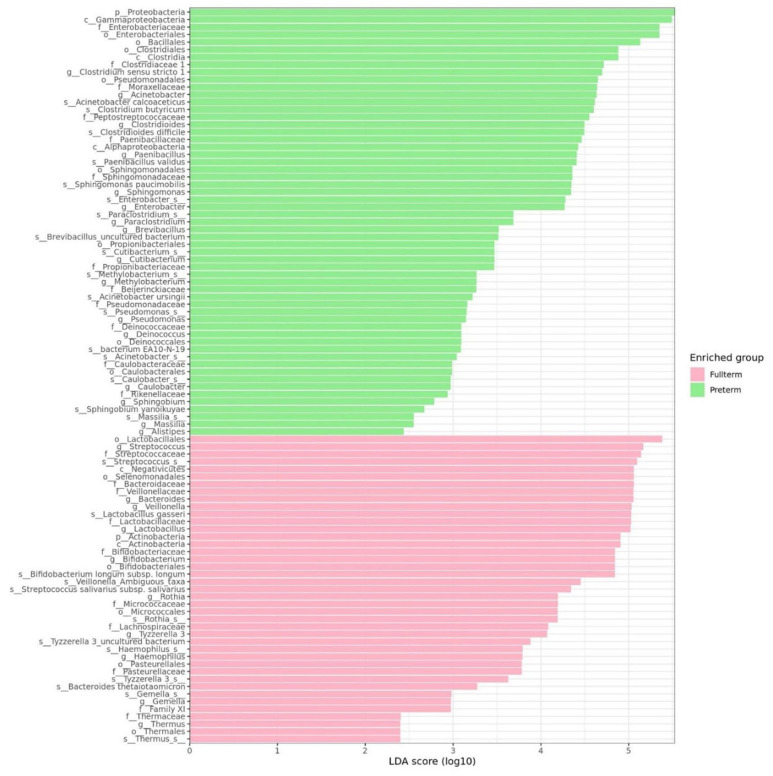
Linear discriminant analysis effect size (LEFse) of full-term and pre-term infants.

Linear discriminant analysis effect size (LEFse) analysis is able to determine the distinguishable features of different groups. The full-term group showed biomarkers from the phylum Actinobacteria, especially Bifidobacterium, in addition to members of the phylum Firmicutes and order Lactobacillales, such as Steptococcaceae, as well as Veillonellaceae and the order Actinomycetales, such as Rothia (Figure 6). Conversely, the biomarkers of pre-term infants were mainly dominated by Proteobacteria, especially the potentially pathogenic Enterobacteriaceae and Pseudomonodales, in addition to Clostridiales—mainly Clostridiaceae 1, Clostridium sensu stricto spp., and Peptostreptococcaceae (Figure 6).

**Figure 7:**
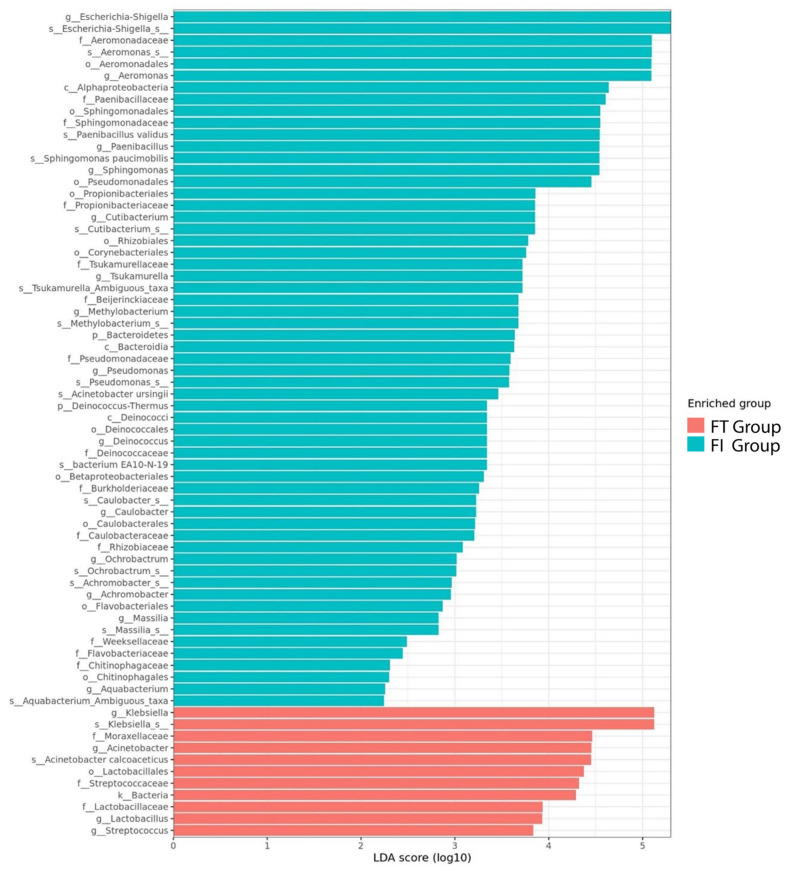
Linear discriminant analysis effect size (LEFse) of infants in the FT and FI groups.

Interestingly, the infants in the FT group shared some biomarkers with the full-term infants (Lactobacillaceae, Streptococcaceae) and their related taxa (Figure 7). On the other hand, the biomarkers of those in the FI group were dominated by the potentially pathogenic Escherichia–Shigella and Sphingomonodaceae, in addition to NICU-associated Aeromonadales and Propionibacteriaceae.

## 4. Discussion

Gut microbiota development in newborns is crucial for their survival and long-term health [20]. Generally, the feeding tolerance of pre-term infants is complicated by dysbiosis and the immature gastrointestinal tract [21,22]. In our study, we observed a higher abundance of gut microbiota in full-term infants, followed by the FT group, while the FI group had the lowest abundance. Secondly, over the first four weeks of life, the microbiome differed in the FT and FI groups, which may result in feeding problems. In the FT group, there were more probiotic biomarkers such as Lactobacillaceae and Streptococcaceae, while more potentially pathogenic *Escherichia*–*Shigella* spp. were detected in the FI group.

We found that in the first four weeks of life, the alpha diversity and abundance of gut microorganisms in the FT group were not only significantly higher than those in the FI group but also continued to change. However, the alpha diversity of gut microorganisms in the FI group did not change significantly at three to four weeks. These findings are consistent with results reported by Hong et al. [23]. In Hong’s study, the microbiomes of infants with FI showed lower alpha diversity and reduced bacterial abundance at three to four weeks of age. Hong et al. proposed that developmental delays in the microbiota during this period may contribute to impaired intestinal function in infants with FI.

The sequential dominance and succession of bacilli (Phase 1, P1), Gammaproteobacteria (Phase 2, P2), and Clostridium (Phase 3, P3) are known to occur in the development of pre-term infants’ microbiota [20,24,25]. P1 is characterized by low levels of initial diversity and abundant facultative anaerobes. This is followed by increased diversity and the blooming of obligate anaerobes to fermentation-based metabolism in P3.

Our FT infants exhibited such progression in the first month of life. Similar to the description of P1, the first week in the FT group was characterized by low diversity and was dominated by the facultative anaerobes Staphylococcaceae and Enterobacteriaceae (Figure 4). In addition, the decrease in the number of bacilli was also demonstrated (Figure 5). The succession to P2 began in the second week of our study, as an increase in the obligate anaerobes Clostridiaceae 1 and Peptostreptococcaceae, along with a drop in the aerobe Moraxellaceae, was observed. In weeks three and four, an increase in Gammaproteobacteria such as Enterobacteriaceae and clostridia including Clostridiaceae 1 and Veillonellaceae marked the transition to P3 (Figure 5).

Such progression was not seen in the FI infants (Figure 4 and Figure 5). Compared to FT, the FI group not only showed less microbiota change in the four weeks after birth but also had lower diversity. The increase in the Firmicutes family member Staphylococcaceae occurred a week later than in the FT group; similarly, no increase in clostridia was observed (Figure 5) in FI, suggesting a progression delay in gut microbiota (Figure 4). Gammaproteobacteria is one class of Proteobacteria, and we can deduce that the P2 is prolonged in the FI group. Grier et al. conducted analyses of the putative functional capacity of the progressive phases, revealing significant roles in the host metabolism and gastrointestinal development.

We considered that the residual oxygen was already used up and the epithelial wall of the gastrointestinal tract was not as permeable to oxygen, allowing obligate anaerobic bacteria to bloom. This indicates the maturation of the pre-term gastrointestinal tract, resulting in FT [26].

Comparing the characteristics of both the FT and FI participants, we found differences in the gestational ages and antibiotics days of the infants. In our study, the FI groups exhibited a longer use of antibiotics and a lower gestational age. According to the literature review, the main driver of microbiota development is gestational age. Antibiotic use had strong but temporary effects, and the birth mode had little influence for the pre-term infants [24,27]. In our study, the top three gut microbiota families in the FI groups were Enterobacteriaceae, Staphylococcaceae, and Aeromonadaceae. Although the participants in the FI groups had a lower gestational age, we did not find the Enterococcus phase, which was only observed among extremely premature infants. Prolonged periods in P1 and P2 may represent the effects of lengthy antibiotic treatment and/or a lack of enteral nutrition. Therefore, our preliminary findings suggest that the gestational age might affect the results. Additionally, we observed a prolonged P2 phase, which could be attributed to perinatal antibiotic treatment. Both factors influence the gut microbiota and potentially contribute to the development of FI.

Proteobacteria were the major biomarker in pre-term infants’ gastrointestinal tracts, while the major biomarkers in the full-term infants’ gastrointestinal tracts mostly constituted Actinobacteria (Figure 6). As a comparison, the aerobes Moraxellaceae and Sphingomonadaceae were practically non-existent in more developed full-term infants (Supplementary Materials Table S2). Both Moraxellaceae and Sphingomonadaceae belong to the phylum Proteobacteria. Especially in the FI group, the biomarkers were associated with Proteobacteria (Figure 7). Among them are *Sphingomonas* (particularly *Sphingomonas paucimobilis*), *Methylobacterium*, Enterobacteriaceae, and *Escherichia-Shigella* spp. *Sphingomonas* and its species *S. paucimobilis* are known to trigger infections in pre-term infants. According to the literature review, total bacterial communities differed significantly between healthy infants and those with NEC or sepsis, with *Sphingomonas* spp. being significantly associated with NEC [28,29]. An abundance of Proteobacteria and low rates of Firmicutes are associated with NEC [30,31].

It is difficult to distinguish between FI and NEC in the early stages. Feeding intolerance (FI) is defined as the inability to digest enteral feedings, and is associated with in-creased gastric residuals, abdominal distension, and/or emesis [9]. However, NEC, a devastating intestinal disease that affects premature infants, presents as lethargy, apnea, bradycardia, thermic instability associated with biliary gastric residues, vomiting, and abdominal distension with/without rectal bleeding [32]. Many studies suggest that gut microbiota dysbiosis may be associated with NEC. Modified Bell staging remains the most commonly utilized case definition of NEC worldwide [33]. We compared our study results to the findings related to suspected NEC (NECI) from a case–control study [34]. In Brehin’s study, there was a higher abundance of *streptococcus* species in the second 10 days of life, followed by *Staphylococcus* species in the third 10 days of life in NEC I. Meanwhile, the abundance of Staphylococcaceae in the FI group remained consistent during the first month of life. Even in the FI groups, we provided more antibiotic days, and we did not observe delayed intestinal colonization by Proteobacteria but prolonged the P2 to P3. Brehin et al. [34] concluded that NECI gut microbiota appear more distinguishable by the third ten days of life; this finding is consistent with our finding that abundant Staphylococcaceae might induce FI. The risk of major complications associated with very pre-term birth, including necrotizing enterocolitis (NEC) [35], late-onset bacterial infections [36], and post-natal growth impairment [37], have been linked to alterations in gut microbiota composition that occur prior to these complications [38]. To the best of our knowledge, this is the first study to use 16S rRNA sequencing to investigate the connection between the feeding problems of pre-term infants and the functionality of the gut microbiota composition.

The first limitation of this study is that there are several factors that affect the gut microbiota, including environmental and genetic factors. For example, during pregnancy, the main influencing factors of the maternal microbiota are diet, use of antibiotics, infection, stress, and host genetics. During delivery, delivery mode and the gestational age of the newborn at birth affect the colonization of the newborn’s microbiota [39]. In our study, due to the small number of participants, we collected the parameters to evaluate group dissimilarities instead of using a paired-control approach. Because of the resource constraints, we did not conduct a power analysis and instead enrolled as many participants as possible within the limited timeframe. Meanwhile, the main influencing factor of the infant microbiota is the feeding mode. It is difficult to ensure that all participants underwent breast milk feeding throughout the whole study period. We ensured that the feeding mode was as similar as possible across the three groups. All enrolled infants had at least two weeks of breast milk feeding. Secondly, dysbiosis, or microbial imbalance, is linked to a number of diseases in infants such as asthma, Crohn’s disease, inflammatory bowel disease (IBD), necrotizing enterocolitis, and type 1 diabetes (T1D) [40]. However, we had no weekly data for the metabolic pathway when there was a delay in the gut microbiota maturation and no clinical issues that matched with some of the pathways. Therefore, we cannot determine the timing of inoculation and can only demonstrate that the infants in the FI group exhibited a higher prevalence of disease-related pathways. Further study should be carried out.

## 5. Conclusions

A delayed maturation in the gut microbiota might result in feeding intolerance in pre-term infants. Greater consideration is required when using antibiotics in clinical practice.

## Figures and Tables

**Table 1:** Analysis of similarities (ANOSIM) values of pre-term vs. full-term infants and FT vs. FI pre-term infants.

Grouping	R Statistic Value ^1^	*p* Value
Pre-term vs. full-term	0.2955	0.0001
FT vs. FI	0.1733	0.0009
FT Group		
Week 1 vs. 2	0.3188	0.0160
Week 1 vs. 3	0.5556	0.0013
Week 1 vs. 4	0.5251	0.0019
Week 2 vs. 3	−0.1453	0.9499
Week 2 vs. 4	−0.0836	0.7585
Week 3 vs. 4	−0.0651	0.7016
FI Group		
Week 1 vs. 2	0.3125	0.0400
Week 1 vs. 3	−0.1440	0.9933
Week 1 vs. 4	−0.0720	0.7285
Week 2 vs. 3	−0.0992	0.6953
Week 2 vs. 4	−0.0714	0.6192
Week 3 vs. 4	−0.1448	0.8866

^1^ An R statistic value >0 means that the difference between groups is significant; <0 means the difference within the group is greater than the difference between groups. A *p* value <0.05 indicates statistical significance.

## Data Availability

Results of DNA sequencing are available on NCBI SRA (Submission ID: SUB14221132; BioProject ID: PRJNA1077685).

## Supplementary Materials

The following supporting information can be downloaded at: https://www.mdpi.com/article/10.3390/biomedicines12030539/s1, Table S1: Demographic Data, Figure S1: Flow chart of participants recruitment, Table S2: Absolute abundance of Moraxellaceae in the full-term and pre-term groups.
