# Supplementary material for: The Association between Delayed Gut Microbiota Maturity in Pre-Term Infants and the Feeding Intolerance—A Pilot Study
Source: Biomedicines. 2024 Feb 28;12(3):539. doi: 10.3390/biomedicines12030539 (PMC10967967; doi:10.3390/biomedicines12030539)
Supplement: Supplementary file 1 [file biomedicines-12-00539-s001.zip › biomedicines-2833688-supplementary.pdf]

Table S1. Demographic data.

characteristics	Full-term infants (n=10)	Preterm infants (n=13)		
		Feeding tolerance (FT) group (n=7)	Feeding intolerance (FI) group (n=6)	P value
**Infant characteristics**	Median (Minimum, Maximum) or n			
Gestational Age (week)	38.07 (37, 40.29)	32.0 (30.0, 35.42)	29.14 (27.57, 32)	0.192
Birth Weight (gm)	3170 (2628, 3450)	1680 (1250, 2290)	1215 (1020, 1710)	0.592
Antibiotic Day[a]	0	3 (3, 7)	8.5 (3, 14)	0.029*
NEC	0	0	0	
Blood ABG[b]	N/A	7.347 (7.24, 7.51)	7.362 (7.32, 7.42)	1.00
**Type of Delivery**				
Spontaneous Delivery	7	2	1	1.00
C section	3	5	5	
**Mother characteristics**	Median (Minimum, Maximum) or n			
**GDM**	0	1	1	1.00
**PPROM**	0	3	4	0.592
**BMI**	26.19 (21.72, 31.79)	26.36 (21.87, 31.89)	23.89 (22.2, 30.48)	0.592
**Antibiotic Day[c]**	0	0 (0, 9)	0 (0, 9)	0.559

a. The duration of antibiotic usage in the first 14 day of life
b. The first blood sampling after birth
c. The duration of antibiotic usage in mother admission history 14 days prior to birth
d. NEC = necrotizing enterocolitis, ABG = arterial blood gas, N/A=Not Applicable, GDM = gestational diabetes mellitus, PPROM= preterm premature rupture of membrane; BMI= body mass index

Figure S1. Flow chart of participants recruitment.

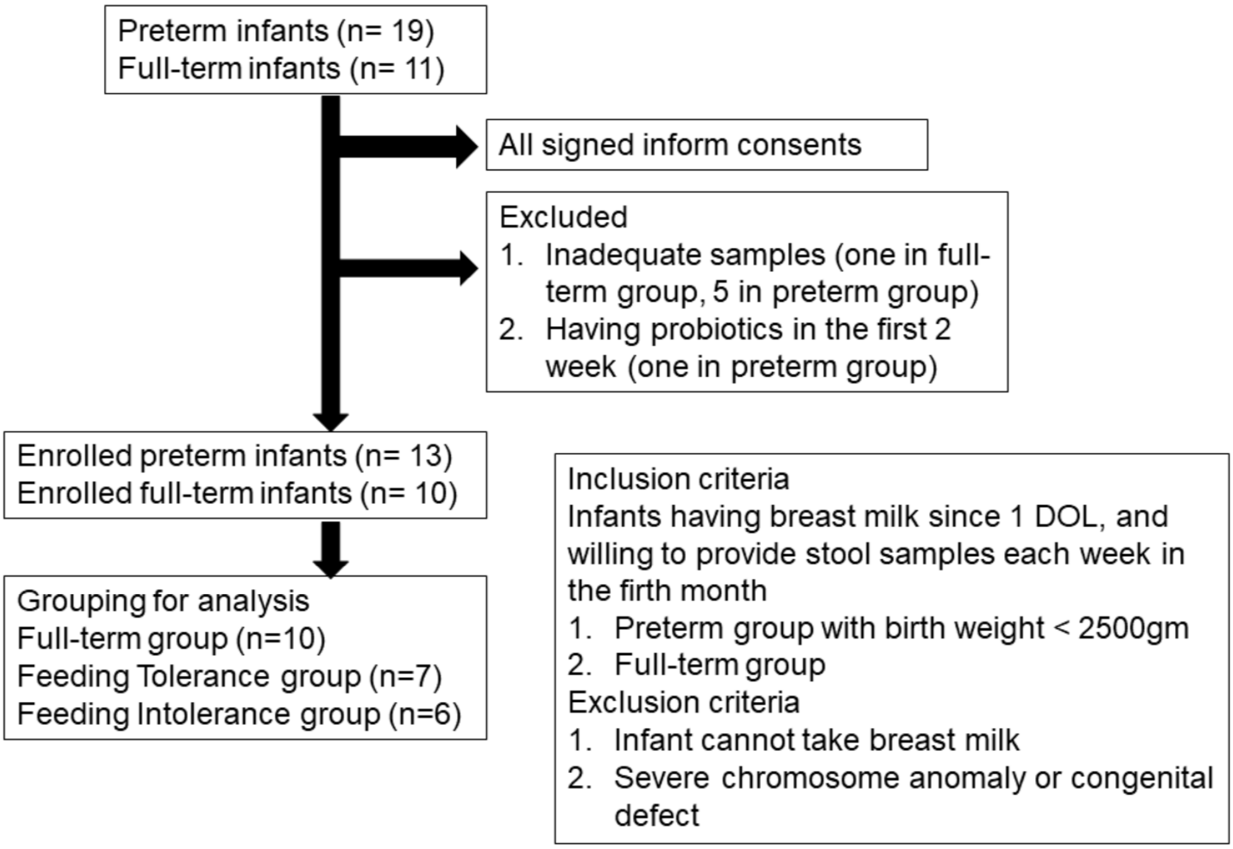

Table S2. Absolute abundance of Moraxellaceae in full-term and preterm group.

	**Full-term (n=10)**	**Preterm (n=13)**
**Total read**	2026599	2498519
Moraxellaceae	1845	83953
